# A monoclonal natural human IgM protects axons in the absence of remyelination

**DOI:** 10.1186/s12974-016-0561-3

**Published:** 2016-04-28

**Authors:** Bharath Wootla, Aleksandar Denic, Arthur E. Warrington, Moses Rodriguez

**Affiliations:** Department of Neurology, Mayo Clinic College of Medicine, Mayo Clinic, 200 First Street SW, Rochester, MN 55905 USA; Mayo Clinic Center for Multiple Sclerosis and Autoimmune Neurology, Mayo Clinic, 200 First Street SW, Rochester, MN 55905 USA; Center for Regenerative Medicine, Neuroregeneration, Mayo Clinic, 200 First Street SW, Rochester, MN 55905 USA; Department of Immunology, Mayo Clinic College of Medicine, Mayo Clinic, 200 1st Street SW, Rochester, MN 55905 USA

**Keywords:** Multiple sclerosis, Theiler’s murine encephalomyelitis virus, Brainstem, Axons, Retrograde labeling, Activity monitoring

## Abstract

**Background:**

Whereas demyelination underlies early neurological symptoms in multiple sclerosis (MS), axonal damage is considered critical for permanent chronic deficits. Intracerebral infection of susceptible mouse strains with Theiler’s murine encephalomyelitis virus (TMEV) results in chronic induced demyelinating disease (TMEV-IDD) with progressive axonal loss and neurologic dysfunction similar to progressive forms of MS. We previously reported that treatment of chronic TMEV-IDD mice with a neurite outgrowth-promoting natural human antibody, HIgM12, improved brainstem NAA concentrations and preserved functional motor activity. In order to translate this antibody toward clinical trial, we generated a fully human recombinant form of HIgM12, rHIgM12, determined the optimal in vivo dose for functional improvement in TMEV-IDD, and evaluated the functional preservation of descending spinal cord axons by retrograde labeling.

**Findings:**

SJL/J mice at 45 to 90 days post infection (dpi) were studied. A single intraperitoneal dose of 0.25 mg/kg of rHIgM12 per mouse is sufficient to preserve motor function in TMEV-IDD. The optimal dose was 10 mg/kg. rHIgM12 treatment protected the functional transport in spinal cord axons and led to 40 % more Fluoro-Gold-labeled brainstem neurons in retrograde transport studies. This suggests that axons are not only present but also functionally competent. rHIgM12-treated mice also contained more mid-thoracic (T6) spinal cord axons than controls.

**Conclusions:**

This study confirms that a fully human recombinant neurite outgrowth-promoting monoclonal IgM is therapeutic in a model of progressive MS using multiple reparative readouts. The minimum effective dose is similar to that of a remyelination-promoting monoclonal human IgM discovered by our group that is presently in clinical trials for MS.

## Findings

### Introduction

Multiple sclerosis (MS) is an inflammatory demyelinating disease of the central nervous system (CNS). Axonal damage is considered critical for permanent chronic deficits in progressive MS, but the precise mechanisms by which axonal injury occurs in MS are unclear. None of the currently FDA-approved drugs or candidates in clinical trial protect neurons and axons from degeneration. Theiler’s murine encephalomyelitis virus (TMEV) is a single-stranded RNA virus that belongs to the Picornaviridae family. Intracerebral injection of TMEV induces an inflammatory demyelinating disease in the spinal cord of susceptible strains of mice following infection [1]. TMEV-induced demyelinating disease (TMEV-IDD) is a natural chronic progressive CNS demyelinating disease of susceptible strains of mice, with similarities to primary progressive MS [2]. The TMEV-induced model of demyelinating disease is a reasonable platform for therapeutic drug discovery for progressive forms of demyelination.

We previously reported that a human IgM monoclonal antibody, HIgM12, bound to the surface of neurons, supported robust neurite extension when presented as a substrate, and overrode the neurite extension inhibition of CNS myelin and myelin-associated glycoprotein (MAG) [3–5]. We also showed that a single 10 mg/kg dose of HIgM12, when peripherally administered, improved clinical disease course of chronic virus-infected mice beginning 2 weeks following treatment, which persisted for 8 weeks [6]. In a recent follow-up study, HIgM12, when administered intraperitoneally to 90-day TMEV-IDD mice, improved brainstem NAA concentrations, a surrogate biomarker for the density of spinal cord axons, at 5 and 10 weeks following treatment [7]. This improvement was present even though HIgM12 does not promote spinal cord remyelination. A reagent that protects axons without associated remyelination would be a critical drug by itself or paired with immune-modulatory or remyelination-promoting therapies. To translate this IgM to clinical trial, we generated a fully human recombinant form of the IgM (rHIgM12) from CHO cells and tested this for efficacy in the TMEV model. We first determined the minimum and optimum dose of rHIgM12 that improves neurologic function in vivo by locomotor monitoring of TMEV-IDD mice.

Active axonal retrograde labeling relies on both anatomically continuous axons and preserved retrograde transport mechanism which provides an assessment of axonal integrity. Retrograde labeling analysis of spinal cord axons in chronically demyelinated mice previously revealed a marked reduction in the number of labeled neuronal cell bodies in the brainstem [8]. Our recent report [7] describing an increase in brainstem NAA concentrations after treatment with HIgM12 led us to determine whether treatment with rHIgM12 results in an increased number of retrograde tracer-positive neuron cell bodies within the brainstem, indicating functional transport.

## Methods

### Ethics statements

The Mayo Clinic Institutional Animal Care and Use Committee (IACUC) approved all animal protocols used in this study.

### Theiler’s virus model of demyelination

Demyelinating disease was induced in 8-week-old SJL/J mice by intracerebral injection of 10 μl containing 2.0 × 10^5^ plaque-forming units (PFUs) of Daniel’s strain TMEV [9]. This typically results in >98 % incidence of infection with rare fatalities.

### Antibodies

**Fig. 1:**
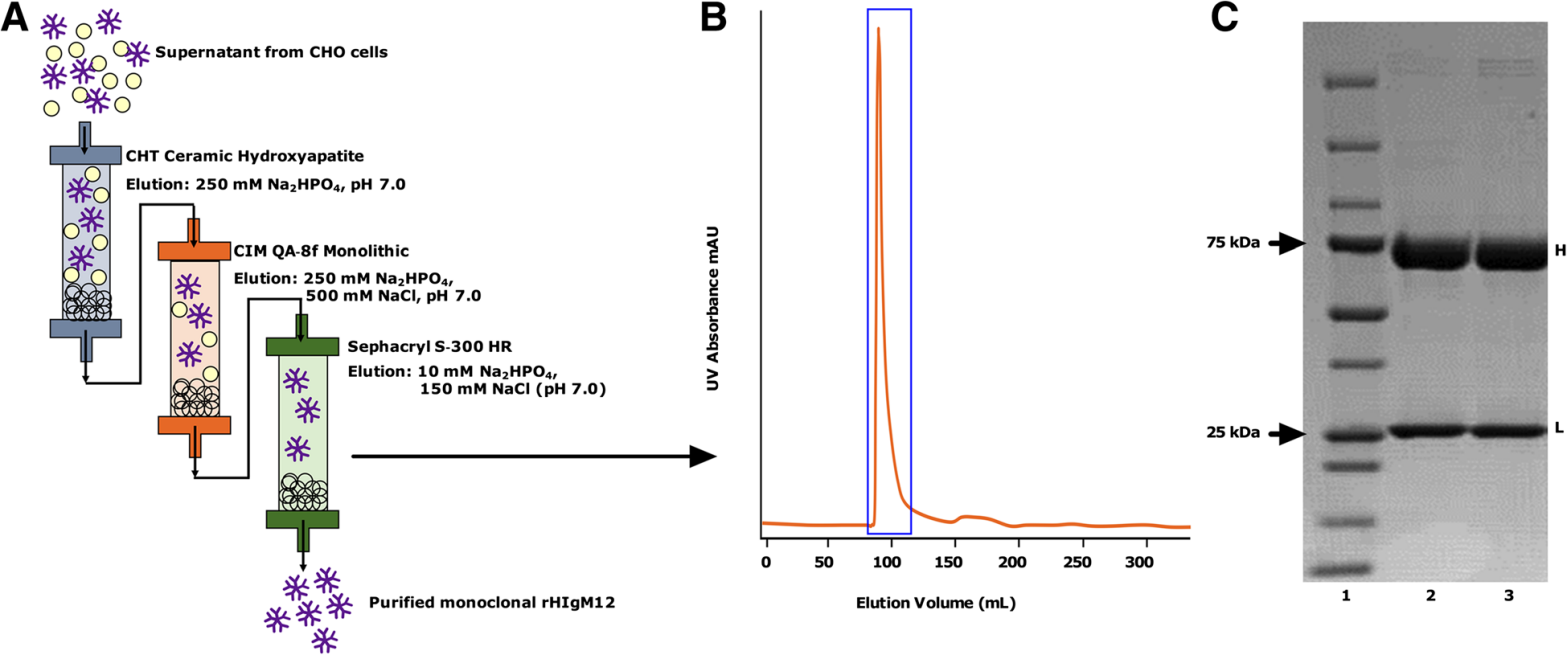
Purification of rHIgM12 to >97 %. IgM was isolated from culture supernatant using a three-step purification protocol. **a** shows a flow diagram of the three-step purification protocol. **b** shows the final eluate from the third step, i.e., separation on a Sephacryl S-300 HR column in the presence of 10 mM NaOH and 150 mM NaCl (pH 7.0). **c** shows an SDS-PAGE gel of the following: *lane 1*—molecular weight markers, *lanes 2 and 3*—20-μl samples of the peak fraction showed in **b**. The IgM heavy chain (*H*, 75 kDa) and light chain (*L*, 25 kDa) are shown

Human IgM12 was identified and isolated from a patient (N°12) with Waldenstrom’s macroglobulinemia carrying high levels of this monoclonal protein for years without detriment. The variable region amino acid and nucleotide sequences for the heavy and light chains are available in GenBank under the accession numbers DQ146928 and DQ146929, respectively. rHIgM12 was expressed in CHO-S cells (GibcoBRL, cat# 11619). Plasmids expressing the heavy and light chain coding sequences were co-transfected along with a human J chain. The resulting cells were selected with increasing doses of methotrexate, and clones that produced IgM as measured by ELISA were sub-cloned and expanded. A stable clone was expanded, banked, and successfully grown in hollow fiber or wave bags to obtain supernatant. rHIgM12 was purified in a qualified GLP facility. Briefly, diafiltered culture supernatant containing rHIgM12 was subjected to a three-step purification protocol (Fig. 1a). First, culture supernatant was loaded on a CHT Ceramic Hydroxyapatite, Type II (Bio-Rad) column and eluted using 250 mM sodium phosphate (NaOH, pH 7.0). The eluted solution was then passed through a CIM QA-8f Monolithic column (BIA Separations) followed by elution with 250 mM NaOH, 500 mM NaCl (pH 7.0). In the final step of purification, the eluate from the second step was further subjected to separation on a Sephacryl S-300 HR column in the presence of 50 mM Na_2_HPO_4_, 150 mM NaCl (pH 7.0). Collection of only peak fractions at the final step allowed approximately 6-fold concentration of the eluate from the monolith column. The purity of eluates at each step is shown (Fig. 1b, c). The stock rHIgM12 preparation was stored as 5 mg/ml aliquots. The final IgM was validated using titrated potency assays of binding to the surface of neurons and support of neurite extension. The potency of rHIgM12 and the serum-derived IgM were compared [10] and found to be identical therapeutically. rHIgM12 can be obtained for non-commercial experimental use by contacting the corresponding author. The control human IgM used was isolated from human serum [4]. It did not bind to neurons in culture or live tissue slices nor support neurite extension in cell culture. Informed consent was obtained from all patients for the use of serum samples for research purposes.

### Treatment of mice

Dosing study: SJL mice at 45 days post infection (dpi), the time point when demyelination in the spinal cord is well established and before substantial axon loss, were treated with a single intraperitoneal dose of rHIgM12 (0.25, 2.5, 10, or 25 mg/kg) or isotype control IgM (10 mg/kg). Dosing was based on the average weight of an adult SJL mouse (20 g). Retrograde labeling study: SJL mice at 90 dpi, the time point when axonal degeneration is present and progressing, were treated with a single intraperitoneal dose of rHIgM12 (10 mg/kg) or saline.

### Spontaneous activity monitoring

**Fig. 2:**
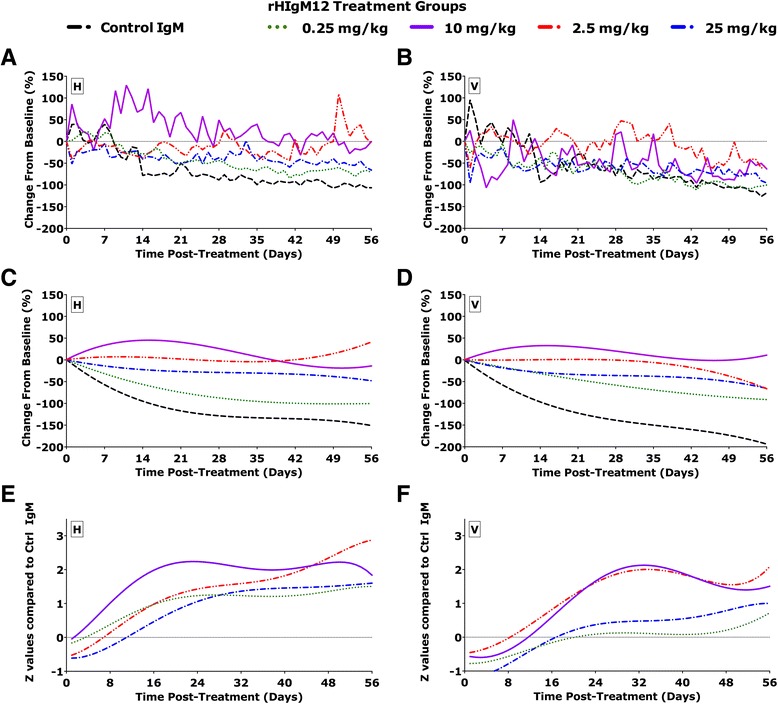
rHIgM12 treatment improves both horizontal and vertical activity in SJL mice during established demyelinating disease. Groups of SJL mice (*N* = 10 per treatment group) at 45 dpi were placed in activity monitoring boxes. Baseline measurements were collected over 8 days. Beginning at treatment (time zero), mice were monitored continuously over an additional 8 weeks. **a**, **c** correspond to horizontal activity and **b**, **d** correspond to vertical activity. **a**, **b** Original, unfiltered recordings for horizontal and vertical activity normalized to baseline; **c**, **d** third-order polynomial fitting of standardized *z* values normalized to baseline revealed improvement in both horizontal and vertical activity of the rHIgM12-treated group compared to the control IgM-treated group. Horizontal (**e**) and vertical (**f**) nocturnal activity of rHIgM12-treated mice compared to control IgM-treated mice significantly diverged above the *x*-axis (more activity) (*p* < 0.05) post-treatment. The pointwise lower 95 % confidence bands for the nocturnal activity are represented on the *y*-axis scale

Spontaneous horizontal and vertical locomotor activity was recorded using open-field (OF) locomotor activity monitors (Omnitech Electronics; Columbus, OH) and Fusion v5.0 software. Each monitor consists of sets of 16 light beam arrays in the horizontal *x* and *y* axes. The hardware detects beams broken by animal movements to determine the location within the cage. In all cages, mice were exposed to identical environmental conditions: (a) freely accessible food and water; (b) a normal 12-h light/dark cycle; and (c) 70 °F ambient temperature. Five SJL mice at 45 dpi were placed in each cage, and baseline spontaneous activity was collected over a period of 5 consecutive days. Groups of mice were then treated with a single dose of rHIgM12 (0.25, 2.5, 10, or 25 mg/kg) or with 10 mg/kg of control human IgM antibody. Following treatment, mice were continuously monitored for 56 days. The total horizontal and vertical activity data, quantified as mean hourly mean breaks, was exported to an Excel (Microsoft Corporation) compatible file for further analysis. The original activity box data sets were first normalized to baseline activity independently for each group of mice (Fig. 2a, b) followed by a polynomial curve fitting (Fig. 2c, d). We described this methodology in greater detail (see [6]). Briefly, the model was designed to allow for polynomial terms up to any degree (xn) and estimated shape parameters separately for each dose and treatment group. For the analysis of datasets in this study, we chose the third-degree polynomial followed by normalization of curves to *y*-axis = 0. This allowed us sufficient flexibility to model non-linear effects over time. Polynomial analysis of data sets was considered ideal as it allowed us to perform direct pairwise comparison at regular intervals across the entire time frame to determine when treatment groups have significantly diverged. In addition, a polynomial fit clarifies data by eliminating noise to draw visual attention on the general trend over the entire time frame. Polynomial regression models were performed and plotted using Graphpad Prism v5.0.

### Retrograde labeling

Retrograde labeling [8] of brainstem neurons was performed on 23 mice (uninfected = 3, rHIgM12 = 9, saline = 11) at 9 weeks after antibody treatment. Briefly, dorsal laminectomy of the lower thoracic vertebrae was performed on anesthetized mice. The spinal cord was hemisected on the right side using microdissecting blades. The retrograde tracer, Fluoro-Gold, was applied and the surgery site was closed. One week post-surgery (i.e., 10 weeks post-treatment), mice were terminally anesthetized, perfused with 4 % paraformaldehyde, and brains and spinal cords were collected. Serial vibratome (Lancer Series 1200) sections (40 μm thick) of the brainstem were collected and mounted under Vectashield (Vector Laboratories Inc., Burlingame, CA). Cell bodies containing retrograde tracer (Fluoro-Gold), visualized by UV illumination (360–370-nm excitation, 420–460-nm emission) were counted at 200× from 16 brainstem slices per mouse. A cell was counted as positive if a large cross-section of the cell body was labeled with Fluoro-Gold. All analyses were performed without knowledge of the experimental groups.

### Spinal cord morphometry

We assessed spinal cord morphometry in all groups 10 weeks following treatment. Mice were anesthetized with sodium pentobarbital and perfused intracardially with Trump’s fixative (phosphate-buffered 4 % formaldehyde/1 % glutaraldehyde, pH 7.4). Spinal cords were removed and sectioned precisely into 1-mm blocks. In order to represent samples along the length of the spinal cord, every third block was postfixed, stained with osmium tetroxide, and embedded in araldite plastic (Polysciences, Warrington, PA). One-micrometer sections were cut and stained with 4 % p-phenylenediamine to visualize the myelin sheaths. We examined ten spinal cord cross-sections, spanning the entire spinal cord from cervical to the distal lumbar regions, from each mouse. Each spinal cord quadrant from every coronal section was graded for the presence of inflammation and demyelination. Quadrants with inflammation were defined by the presence of inflammatory cells lining the meninges. Areas of demyelination were defined as containing naked axons, macrophage infiltration, myelin ovoids, and degenerated axon profiles. These areas were well demarcated and allow accurate quantitative assessment at 10× and 40× magnifications, respectively. Demyelination or inflammation scores were expressed as the percentage of spinal cord quadrants examined with pathological abnormality. A maximum score of 100 indicated pathological abnormality in every quadrant of all spinal cord sections of a given mouse. All grading was performed on coded sections without knowledge of the experimental group.

To quantify myelinated axons, a mid-thoracic (T6) spinal cord section from each animal was examined. This level of the cord was chosen because it contains both ascending axons and descending axons some of which have neuronal bodies in the brainstem [11]. To ensure a uniform intensity of myelin labeling, all spinal cord T6 sections used in the study were stained with the same batch of 4 % para-phenylenediamine for exactly 20 min. An Olympus Provis AX70 microscope and a 60× oil-immersion objective were used to capture six sample areas of normal-appearing white matter without demyelination from each section. The fields were collected in a clockwise manner around the section to obtain representative samplings of the posterior-lateral, antero-lateral, and anterior columns. Images were centered between the gray matter and meningeal surface. Approximately 400,000 μm^2^ of white matter was sampled from each mouse. Absolute myelinated axon numbers were measured using automated counting software that recognizes circular intact myelin sheathes and calculated as reported [12]. Data were represented as the absolute number of all axons sampled per mid-thoracic spinal cord section. All values were averaged per group.

### Statistics

Data from activity monitoring: Statistical comparisons of treatment groups were performed using the predicted model values and respective standard errors based on the *z*-statistic (SAS Institute, Inc.). Direct pairwise comparisons of treatments were performed for each day across the entire time frame, and statistical significance was determined at the typical *a* = 0.05 threshold. No adjustments were made for multiple comparisons. Data for retrograde labeling and axon-count analysis were compared by Student’s *t* test if normally distributed or by Mann-Whitney rank sum test if non-normally distributed. In all analyses, *p* < 0.05 was considered as statistically significant. Correlation coefficients between paired sets of data were determined using the Pearson product moment correlation.

## Results

Nocturnal behavior is a sensitive measure of neurologic deficits in TMEV-IDD [6]. In this study, we used this assay to determine the minimum and the most effective dose of rHIgM12 that improves spontaneous horizontal and vertical locomotor activity in TMEV-IDD. To address this, we monitored 45 dpi TMEV-IDD mice (*N* = 10 two groups of 5 mice per treatment) treated with either rHIgM12 at 0.25, 2.5, 10, or 25 mg/kg or control human IgM (10 mg/kg). Following treatment, spontaneous activity was recorded continuously over 8 weeks. The original raw activity data is noisy (Fig. 2a, b). In order to allow a better visual comparison, we subjected the data to third-degree polynomial curve fitting of standardized *z* values (Fig. 2c, d). This allowed a visual comparison of groups. Using direct pairwise comparisons (Fig. 2e, f) of activity after polynomial fitting, we determined that improved horizontal nocturnal motor function in rHIgM12-treated mice became statistically significant at days 6, 9, 3, and 14 post-treatment for the 0.25-, 2.5-, 10-, and 25-mg/kg doses, respectively, as compared to control IgM (Fig. 2e). Improvement in horizontal nocturnal activity of rHIgM12-treated animals persisted until the end of experiment at 8 weeks. Improved vertical nocturnal motor function in rHIgM12-treated mice became statistically significant at days 12, 15, and 23 post-treatment for the 2.5-, 10-, and 25-mg/kg doses, respectively, as compared to control IgM (Fig. 2f). Vertical activity in the 0.25-mg/kg dose group was not statistically significant at any time point post-treatment when compared to control IgM.

**Fig. 3:**
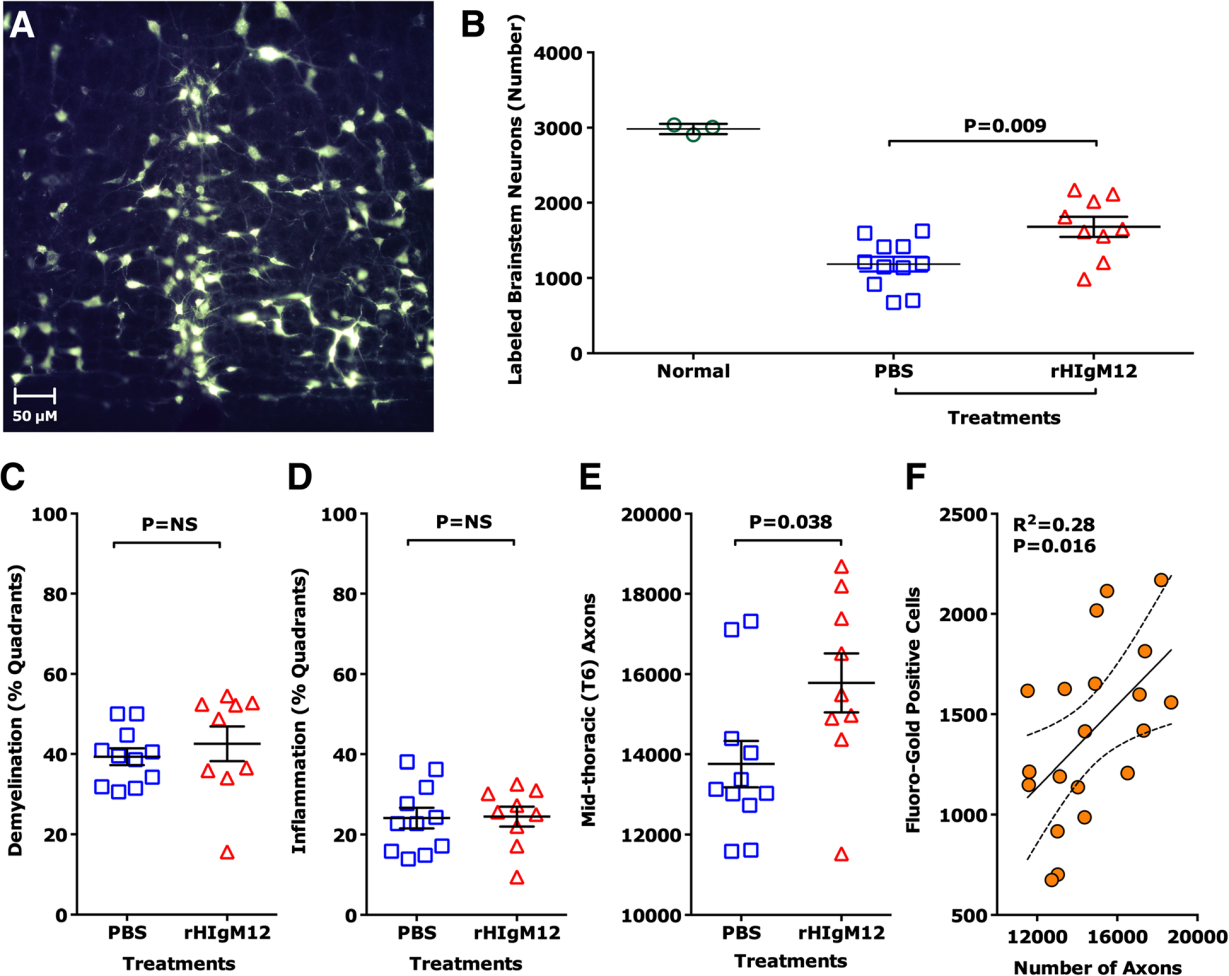
rHIgM12 treatment improves the number of retrograde-labeled brainstem neurons and preserves spinal cord axons, but does not affect spinal cord demyelination. **a** Fluoro-Gold-labeled neurons were counted in brain stem sections. The panel shows an example of a cluster of fluorescently labeled neurons in the brainstem. Extensive labeling of cell bodies as well as axons and dendrites can be easily appreciated. **b** rHIgM12 treatment increased the number of retrograde-labeled brainstem neurons compared to the saline-treated group (*p* = 0.009, Mann-Whitney rank sum test). The number of labeled neurons in uninfected positive control mice (*N* = 3, *circles*) is shown for reference. Forest plots show the average number of retrograde-labeled brainstem neurons ± SEM per treatment group: rHIgM12 (*red triangles*) and saline (*blue boxes*). Mice from both treatment groups had similar levels of spinal cord **c** demyelination and **d** inflammation pathology. Pathology analysis was performed blinded. **e** When the number of myelinated mid-thoracic-level spinal cord axons was compared between treatment groups, rHIgM12-treated mice contained 14.7 % more axons than the saline-treated group (*p* = 0.038, Mann-Whitney rank sum test). **f** The number of fluorescent retrograde-labeled brain stem neurons in each mouse correlated positively and significantly with the number of thoracic-level myelinated spinal cord axons (*p* = 0.016, *R* ^2^ = 0.28)

We recently reported that treatment of TMEV-infected SJL mice with the myelin/oligodendrocyte-reactive human IgM, rHIgM22, resulted in more retrogradely labeled neuronal cell bodies in the brainstem indicating that improving the level of remyelination can preserve function in spinal cord axons [13]. We used the same retrograde labeling assay to investigate whether treatment with rHIgM12, which does not improve the levels of remyelination, could directly protect neurons in the brain stem and spinal cord axons. Functional preservation of spinal cord axons may underlie rHIgM12 improvement of brainstem NAA concentrations [7] and locomotor activity. Retrograde labeling relies on both anatomically continuous axons and preserved retrograde transport mechanisms. We established TMEV-IDD in 20 susceptible SJL mice. Nine mice were treated at 90 dpi with 10 mg/kg of rHIgM12; the remaining 11 mice were administered through vehicle. At 9 weeks post-treatment, we performed retrograde labeling on all 23 mice (uninfected = 3, rHIgM12 = 9, saline = 11). Figure 3a shows an example of a cluster of fluorescently labeled neurons in the brainstem, where cell bodies as well as dendrites and axons are clearly seen. For each descending neuron population, cell bodies containing retrogradely transported Fluoro-Gold label were quantified. Uninfected mice (*N* = 3) had an average number of 2983 ± 39 (mean ± SEM) labeled brainstem neurons. Retrograde labeling studies performed in demyelinated mice demonstrated a large reduction in fluorescently labeled neuron cell bodies in the brainstem [8]. When we quantified fluorescently labeled brainstem neurons in TMEV-IDD mice, as expected, we found fewer labeled neurons compared to uninfected mice. We counted on average 1185 ± 98 labeled neurons in the control-treated animals (*N* = 11) and 1682 ± 134 labeled neurons in rHIgM12-treated animals (*N* = 9). The difference in labeled neuron numbers between the rHIgM12-treated group compared to the saline-treated group was significantly different (*p* = 0.009, Mann-Whitney test, two-tailed) (Fig. 3b).

We investigated the spinal cord pathology at 10 weeks post-treatment. Ten plastic-embedded cross-sections encompassing the entire length of the cord from each mouse were scored for demyelination (Fig. 3c) and inflammation (Fig. 3d). Pathological scores were similar between both groups of mice. We then determined the number of intact axons present in mid-thoracic (T6) spinal cord sections. Six areas encompassing approximately 400,000 μm^2^ of white matter were systematically sampled from each mouse, and the total number of mid-thoracic axons was compared across treatment groups (Fig. 3e). We found more axons in the rHIgM12-treated group compared to the saline-treated group (15,782 ± 736 vs 13,758 ± 575, *p* = 0.038). When the quantity of retrogradely labeled brainstem neurons was plotted as a function of the number of intact myelinated axons at the T6 level from all animals, irrespective of the treatment groups (Fig. 3f), we found a positive and significant correlation (*R*^2^ = 0.28; *p* = 0.016).

## Discussion

There is much evidence to support the goal of remyelination as a means to prevent axon degeneration and slow deficit progression in neurologic disease [14]. In the year 2000, we reported the identification of a natural human IgM that promoted robust spinal cord remyelination in both the TMEV-IDD and lysolecithin-induced demyelination models [15, 16]. A recombinant form of this human IgM, termed rHIgM22, was expressed in a F3B6 cell line with the assembled IgM containing a mouse J chain [17]. rHIgM22 binds to myelin and the surface of oligodendrocytes (OL) and in pre-clinical studies is effective in vivo at very low doses. A single 0.025 mg/kg intraperitoneal injection of rHIgM22 given to TMEV-IDD mice with demyelination promoted significant remyelination 5 weeks later [18] and increased brainstem NAA concentrations [13], indicating a preservation of axon health [19]. In a recently concluded dose escalation clinical trial in humans with MS, rHIgM22 was tested at doses ranging from 0.025 up to 2 mg/kg and found to be completely safe [20].

A second human IgM, rHIgM12, that promotes neurite extension [4], improves brainstem NAA concentrations without associated spinal cord remyelination, suggesting that IgM-mediated neuroprotection can be achieved by an alternate mechanism. In the current study, we determined the minimum and optimum dose of rHIgM12 to preserve spontaneous nocturnal function in groups of TMEV-IDD mice. A dose of 0.25 mg/kg of rHIgM12 improved only horizontal motor function, whereas a dose of 2.5 mg/kg improved both horizontal and vertical activities. An optimum dose of 10 mg/kg provided the best improvement of both horizontal and vertical nocturnal motor function and animals treated with a higher dose of 25 mg/kg did not perform any better. This study suggests that the single dose of rHIgM12 required for biological efficacy in mice is similar to the dose of rHIgM22 required for remyelination. Following identification of the optimum dose, we used the 10 mg/kg dose of rHIgM12 to obtain direct evaluation of descending axon integrity with retrograde labeling at the T6 level of the spinal cord. Our previous work demonstrated that demyelination is normally accompanied by a reduction of axonal transport [8, 21]. The majority of demyelinated lesions in TMEV-IDD occur at the cervical and thoracic levels. Therefore, a reduction in the number of labeled brainstem cells occurs primarily because of disturbed retrograde transport or axonal degeneration. In this study, treatment of SJL mice at 90 dpi with a single dose of rHIgM12 protected the function of spinal cord axons; resulting in 42 % more Fluoro-Gold-labeled brainstem neurons (Fig. 3b) and 14.7 % more axons in the rHIgM12 group compared to the control IgM group. This suggests that a preservation of less than 50 % of brainstem neurons and axons was sufficient to observe functional effects, i.e., an improvement in spontaneous locomotor activity. Our previous magnetic resonance spectroscopy study [7] and neuron/axon-count analyses in the current study provide strong evidence that the neurite outgrowth-promoting antibody, rHIgM12, affected neuronal viability through the preservation of axons. The concept that the degree of axonal damage occurs independently of the extent of chronic demyelination is supported by our data and several other studies that examined gray matter lesions and meningeal infiltrates in human brain and genetically manipulated mice [22–24].

The observed preservation of axons by rHIgM12 may relate to the initially described neurite-promoting activity. Studies of several classic neurotrophins link the promotion of neurite outgrowth with neuroprotection [25, 26]. However, using these pleiotropic molecules in the clinic has been problematic. Antibodies directed against the myelin-derived protein Nogo-A promotes neurite outgrowth in the presence of normally inhibitory molecules [27] and in vivo are therapeutic in models of ALS [28], stroke [29], and spinal cord injury [30]. Treatment of rats with Herceptin, a high-affinity IgG directed against the extracellular domain of the human epidermal growth factor receptor 2 protein (HER2), enhanced axon regeneration after peripheral nerve injury [31]. rHIgM12 binds with high affinity to gangliosides (GD1a and GT1b) [32] and to polysialylated-neural cell adhesion molecule (PSA-NCAM) [33]. GD1a and GT1b are also ligands for the neurite inhibitory molecule myelin-associated glycoprotein (MAG) and facilitate interactions between OLs and axons that maintain long-term axonal stability [34]. MAG-induced inhibition of axon extension involves the rearrangement of neuronal membrane domains and recruitment of p75 neurotrophin receptor to those domains [35]. rHIgM12 binding to the neuronal surface also rearranges plasma-membrane microdomains and clusters signaling molecules, resulting in a shift of microtubule stability and dynamics [36]. We hypothesize that rHIgM12-mediated neurite outgrowth and protection may use the same membrane platforms targeted by antibodies to the neurite outgrowth inhibitor A (Nogo-A) and the leucine rich repeat and immunoglobulin-like domain-containing protein 1 (LINGO-1) [37, 38]. Given that rHIgM12 is produced as a fully human antibody, we expect no toxicity even at higher doses in human studies. Among these neuroprotective reagents, only rHIgM12 was isolated from a human. Most antibody-based therapeutics are humanized forms of originally non-human antibodies. Even humanized antibodies can provoke the synthesis of anti-therapeutic antibodies such as those observed against an anti-CD25 [39] that may limit their long-term effectiveness in humans. Herceptin, used to treat certain breast cancers, carries FDA warnings for cardiomyopathy and embryo-fetal toxicity that limit its widespread use.

Small molecule-based neuroprotective strategies include anti-excitotoxic agents [40, 41], nitric oxide and iNOS inhibitors [42], anti-oxidants [43], Ca^2+^ channel blockers [44], Na^+^ channel blockers [45], Na^+^/Ca^2+^ exchanger inhibitors and growth/neurotrophic factors [46, 47], neural peptides [48], and components of essential oils [49]. A recent study of the MS drug FTY720 reports that this small molecule induces neurite growth, alters growth cone morphology, and promotes axon regeneration [50]. FTY720 treatment of cerebellar neurons induced components of the actin cytoskeleton, which are important for axon growth, but did not alter the levels of tubulin tyrosination or acetylation. In contrast, treatment of cortical neurons with rHIgM12 increased the level of tyrosinated tubulin while decreasing the levels of acetylated tubulin, consistent with promoting a more dynamic cytoskeleton needed for axon growth.

In conclusion, our results provide direct evidence for human antibody-mediated protection of axon integrity in a mouse model of progressive MS. To our best knowledge, rHIgM12 is the first human IgM antibody that is neuroprotective in the absence of remyelination. Combined treatment using both rHIgM22 and rHIgM12 may result in enhanced neuroprotection and better remyelination. However, in TMEV-IDD, complete repair is likely not possible due to the persistent presence of virus. In fact, some of the best histologic repair in TMEV-IDD is driven by treatment with polyclonal human IgM [15], which contains antibodies that bind to both OLs and neurons. A cocktail of human IgMs may be functionally equivalent to this limited resource.

## Abbreviations

CNS: central nervous system
MRS: magnetic resonance spectroscopy
MS: multiple sclerosis
NAA: *N*-acetyl-aspartate
PPMS: primary progressive multiple sclerosis
rHIgM12: recombinant human immunoglobulin M 12
rHIgM22: recombinant human immunoglobulin M 22
RRMS: relapsing remitting multiple sclerosis
SC: spinal cord
SJL: Swiss Jim Lambert
SPMS: secondary progressive multiple sclerosis
TMEV: Theiler’s murine encephalomyelitis virus
